# Predictive Factors for Anastomotic Leakage Following Colorectal Cancer Surgery: Where Are We and Where Are We Going?

**DOI:** 10.3390/curroncol30030236

**Published:** 2023-03-07

**Authors:** Christos Tsalikidis, Athanasia Mitsala, Vasileios I. Mentonis, Konstantinos Romanidis, George Pappas-Gogos, Alexandra K. Tsaroucha, Michail Pitiakoudis

**Affiliations:** 1 Second Department of Surgery, University General Hospital of Alexandroupolis, Democritus University of Thrace Medical School, Dragana, 68100 Alexandroupolis, Greece; 2 Laboratory of Experimental Surgery & Surgical Research, Democritus University of Thrace Medical School, Dragana, 68100 Alexandroupolis, Greece

**Keywords:** anastomotic leakage, colorectal cancer, colorectal surgery, risk factors, anastomotic leakage prediction

## Abstract

Anastomotic leakage (AL) remains one of the most severe complications following colorectal cancer (CRC) surgery. Indeed, leaks that may occur after any type of intestinal anastomosis are commonly associated with a higher reoperation rate and an increased risk of postoperative morbidity and mortality. At first, our review aims to identify specific preoperative, intraoperative and perioperative factors that eventually lead to the development of anastomotic dehiscence based on the current literature. We will also investigate the role of several biomarkers in predicting the presence of ALs following colorectal surgery. Despite significant improvements in perioperative care, advances in surgical techniques, and a high index of suspicion of this complication, the incidence of AL remained stable during the last decades. Thus, gaining a better knowledge of the risk factors that influence the AL rates may help identify high-risk surgical patients requiring more intensive perioperative surveillance. Furthermore, prompt diagnosis of this severe complication may help improve patient survival. To date, several studies have identified predictive biomarkers of ALs, which are most commonly associated with the inflammatory response to colorectal surgery. Interestingly, early diagnosis and evaluation of the severity of this complication may offer a significant opportunity to guide clinical judgement and decision-making.

## 1. Introduction

Colorectal cancer (CRC) is the third most commonly diagnosed malignancy in both men and women worldwide [1]. The rising incidence of colorectal malignancies poses a huge public health concern, as CRC remains a leading cause of cancer-related deaths globally [1]. Meanwhile, based on continuous technological advancements, regular CRC screening has significantly improved clinical outcomes and reduced cancer incidence and mortality [2].

The most common treatment for CRC involves surgical resection of the tumor, which is usually accompanied by a considerable risk of various complications. Anastomotic leakage (AL) represents one of the most severe and feared postoperative complications.

In fact, AL is commonly associated with higher reoperation rates and increased morbidity and mortality risk following colorectal surgery [3,4,5]. The International Study Group of Rectal Cancer (ISREC) defined AL after anterior resection as a defect of continuity localized at the surgical site of the anastomosis, creating communication between intra-luminal and extra-luminal compartments [6]. Interestingly, they proposed three severity grades of this complication, taking into account their impact on the patients’ management [6]. In 2020, an international expert panel of colorectal surgeons and researchers also recommended that the ISREC definition should be considered the generally accepted definition of colorectal AL [7].

Overall, ALs are commonly presented and diagnosed within the first two weeks after surgical intervention [8,9]. Nevertheless, in some cases, a delayed anastomotic leakage could be diagnosed in more than one month following colorectal surgery [8]. Recent studies have presented an incidence of ALs between 2% and 19%, ranging from 2 to 7% when surgery is performed by experienced teams [10,11]. In fact, ALs can occur in up to 24% of patients undergoing surgery for distal rectal cancer [12,13]. This dreadful complication, which mostly leads to increased morbidity and mortality rates, has been associated with higher local recurrence rates and decreased long-term survival [3,4]. Thus far, there is no clear evidence in the literature determining the root cause of ALs [14]. However, most researchers have pointed out several patient-specific, surgical and surgeon-related factors as incriminating in AL pathogenesis after colon and rectal surgery [10,14]. Our review herein aims to help identify all the risk factors related to the development of ALs based on the current literature. In addition, we will present the scoring systems frequently used for predicting ALs and highlight the importance of finding new directions and solutions to reduce the incidence of this feared complication. 

## 2. Risk Factors for Anastomotic Leakage

**Figure 1:**
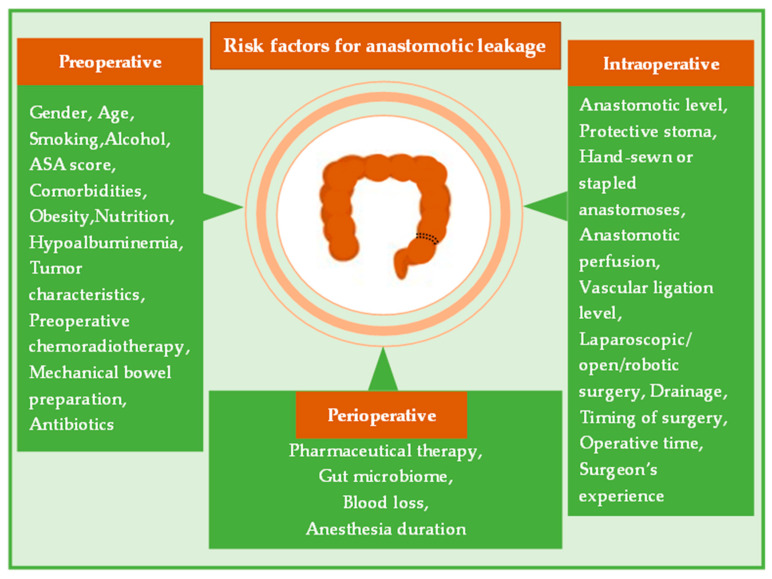
Multiple risk factors have been investigated so far concerning their impact on the incidence of postoperative ALs. Gaining a better knowledge of certain preoperative, intraoperative and perioperative factors could assist the surgeons’ intraoperative judgement and decision-making.

Gaining a better understanding of the potential risk factors that could eventually lead to AL has been the subject of several studies over the past few decades. Thus far, several factors are considered established risk factors, eventually with a predictive value for ALs. However, regarding other potential risk factors, further studies are still required to evaluate their role in developing postoperative AL. To date, multiple risk factors have been identified to affect the incidence of anastomotic dehiscence. These could be classified into preoperative, intraoperative and perioperative, considering different parameters (Figure 1).

### 2.1. Preoperative Risk Factors

Based on the literature, certain factors are considered to have a significant impact on AL rates, either positive or negative. Such factors, mostly patient-related, should be thoroughly examined by the surgeons preoperatively.

#### 2.1.1. Gender and Age

Recent studies have shed light on the relationship between the patients’ gender and the risk of developing AL. Based on comparisons of the incidence rates of postoperative ALs between male and female rectal cancer patients, it has been shown that the male gender represents a significant risk factor for developing this complication [15]. The increased risk of developing ALs in male patients is partially due to technical difficulties and challenges, mainly when a colorectal anastomosis is created in the narrow pelvis [16]. Furthermore, androgen-associated differences between males and females may play a key role in intestinal microcirculation, eventually posing a challenge for colorectal anastomotic healing in males [17].

Novel evidence suggests that pelvic dimensions, particularly pelvic inlet and intertuberous distance, were associated with an increased risk of developing AL after anterior resection [18]. In addition, Verduin et al. [19] revealed that the previously mentioned correlations of male gender and BMI with AL after colon cancer resection were attributed to the visceral fat. Regarding female patients, postoperative AL rates were reduced in those exposed to hormone replacement therapy prior to CRC surgery [20].

To date, several researchers have supported the assertion that male patients are associated with an increased postoperative AL risk. In 2021, Alekseev et al. [21] conducted a study on 429 patients who underwent colorectal surgery and anastomosis. They have shown that the male gender was an independent variable related to higher AL rates (odds ratio (OR) 3.8; 95% confidence interval (CI), 1.9–7.7; *p* < 0.001). Similarly, in another research, Park et al. [15] analyzed 1609 rectal cancer patients and also concluded that the male gender was significantly associated with an increased risk of developing ALs.

Even though mortality following postoperative ALs was significantly associated with the older age of patients [22], some studies found a protective effect of increased patients’ age on AL rates [22,23]. Boström et al. [24] supported the assertion that there was an interaction between AL and age after anterior resection (*p* = 0.007), mainly contributing to increased mortality rates in elderly patients presenting with anastomotic failure. In their prospective study, Lin et al. [25] revealed a significant association between age (>70 years) and AL risk after rectal cancer surgery (*p* = 0.009). Nevertheless, most studies showed that the AL incidence is not correlated with the patient’s age [26,27,28,29,30].

#### 2.1.2. Smoking and Alcohol

Lifestyle factors, including smoking and drinking habits, may play an essential role in colorectal anastomotic healing. Smoking is commonly correlated with an increased AL risk owing to tissue ischemia, mainly due to nicotine-associated vasoconstriction and microvascular disease [31]. It is worth mentioning that heavy smoking history (>40 pack-years) is considered a significant risk factor for developing AL following rectal cancer resection [32]. Furthermore, increased alcohol consumption, particularly exceeding alcohol intake to more than five drinks per day, is also associated with ALs after colorectal surgery [33].

#### 2.1.3. American Society of Anesthesiologists (ASA) Score and Comorbidities

The ASA score is a classification system (I-VI) commonly used to evaluate a patient’s overall health. ASA scoring, combined with other factors, is an effective tool for perioperative risk assessment. Interestingly, patients with an ASA score greater than II were associated with an increased risk of anastomotic dehiscence [30]. In addition, the presence of comorbidities among CRC patients (high Charlson comorbidity index (CCI) score) increases the risk of leaks [30]. Recent research revealed that the ASA score, but not the CCI score, was found to be independently correlated with the development of ALs [34]. Various underlying medical conditions, including diabetes mellitus, renal failure, immunosuppression, pulmonary and cardiovascular disease, were also considered significant risk factors for developing postoperative ALs [35,36,37,38].

#### 2.1.4. Obesity, Nutrition and Hypoalbuminemia

Several studies have shown that obesity is associated with an increased risk of anastomotic failure, especially in patients undergoing low rectal anastomoses. Interestingly, recent research revealed that a body mass index (BMI) > 30 kg/m^2^ was considered an independent risk factor for ALs [39,40,41]. There is also evidence in the current literature to support the assertion that preoperative computed tomography assessment of visceral fat is considered more sensitive than BMI in predicting anastomotic dehiscence after colon cancer surgery [42,43]. In fact, visceral adiposity was found to be related to prolonged operative duration and higher conversion-to-open-surgery, AL and morbidity rates [44].

In addition, malnutrition could contribute to the development of postoperative complications by affecting the process of healing [45]. Several authors support that poor nutritional status may lead to increased complication rates, including anastomotic dehiscence [46]. Indeed, electrolyte imbalance, excessive weight loss and malnutrition are considered to increase the risk of ALs [47]. Furthermore, another study showed that low preoperative levels of serum albumin (<3.5 gr/dL) also appeared to be associated with an increased AL risk in patients suffering from various colorectal pathologies, but mostly CRC and inflammatory bowel disease [48].

#### 2.1.5. Tumor Characteristics

Regarding the characteristics of the tumor, it is widely accepted that the risk of developing AL is correlated with the distal location, large size, advanced stage of colorectal malignancies and the presence of metastatic disease [49]. At first, low distal rectal anastomoses usually have a negative effect on AL rates [49]. The narrow pelvic space and difficult intrapelvic manipulation reflect major challenges in patients undergoing surgery for distal rectal cancer [16]. The distance of the tumor from the anorectal junction is another significant factor affecting the incidence rates of AL [49]. In fact, the tumor’s location in the rectum instead of the colon was independently predictive of anastomotic failure (OR, 18.20; 95% CI, 2.33–142.02; *p* = 0.005) [41]. Tumor size is also considered a contributing factor to AL development. Higher rates of AL are reported for tumors ≥ 3 cm in diameter and a more advanced TNM stage [49,50].

#### 2.1.6. Preoperative Chemoradiotherapy (PCRT)

PCRT is widely used as a part of multimodal treatment strategies, particularly in patients with rectal cancer. Indeed, this approach may improve clinical outcomes by effectively reducing local and distant recurrence rates [51,52]. However, recent research on the incidence of postoperative ALs following PCRT has shown conflicting results. In 2013, Park et al. [15] conducted a retrospective study on 1609 rectal cancer patients after laparoscopic surgery. They found that PCRT was associated with an increased risk of developing anastomotic dehiscence in patients without defunctioning protective stomas (hazard ratio (HR), 6.284; 95% CI, 2.829–13.961; *p* < 0.001). Similarly, other researchers revealed a significant association between increased contained AL rates after rectal cancer surgery and patients who received PCRT (*p* < 0.001) [53].

Regarding treatment characteristics for rectal cancer patients, some authors also supported the assertion that the development of ALs was related to neoadjuvant chemotherapy (*p* = 0.02) [54]. In fact, anastomotic dehiscence was found in 33.3% of patients who received neoadjuvant chemotherapy. Currently, in another research, Qin et al. [55] reported that AL was found in 20.2% of patients who received preoperative radiation and 5-fluorouracil infusion, 23.6% of patients who received the previous therapeutic pattern combined with oxaliplatin, and 8.5% of patients who received preoperative chemotherapy with 5-fluorouracil and oxaliplatin without radiation (*p* = 0.007). The authors revealed a significant association between ALs and preoperative radiation upon multivariate analysis (*p* = 0.02). Results from a large cross-sectional study also showed that neoadjuvant therapy for rectal cancer patients was an independent risk factor for developing ALs during follow-up (OR, 2.85; 95% CI, 1.00–8.11) [56].

Nevertheless, several researchers have supported the assertion that PCRT does not influence the development of ALs. Chang et al. [57] reported that ALs following low anterior resection for rectal cancer patients did not increase in those who received PCRT based on propensity score matching analysis. Another research also showed no statistical difference in the incidence of ALs among low anterior resection patients with and without preoperative radiotherapy [58].

In a meta-analysis of randomized controlled trials, Qin et al. [59] found that preoperative radio(chemo)therapy did not affect the risk of developing ALs after rectal cancer surgery (OR, 1.02; 95% CI, 0.80–1.30; *p* = 0.88). In addition, preoperative radiotherapy or PCRT were not risk factors for postoperative anastomotic failure (OR, 1.04; 95% CI, 0.78–1.39; *p* = 0.78, or OR, 0.98; 95% CI, 0.63–1.53; *p* = 0.94, respectively). Ten randomized controlled trials involving 3951 rectal cancer patients also revealed no significant association between neoadjuvant radiotherapy and ALs (OR, 1.01; 95% CI, 0.82–1.26; *p* = 0.91) [60].

Recently, Hu and colleagues [61] conducted a systematic review and meta-analysis concerning the postoperative AL rates for mid- and low-rectal cancer patients. They revealed that the AL incidence rates were not significantly increased following either short-course (OR, 1.19; 95% CI, 0.89–1.60; *p* = 0.25) or long-course neoadjuvant radiotherapy (OR, 1.38; 95% CI, 0.75–2.54; *p* = 0.31). Furthermore, the interval to surgery after neoadjuvant therapy was not associated with the development of ALs. Meanwhile, another meta-analysis involving rectal cancer patients showed that preoperative radiotherapy and PCRT were correlated with wound complications, such as infection and abscess formation, but not with the postoperative AL incidence [62].

#### 2.1.7. Mechanical Bowel Preparation (MBP) and Antibiotics

Overall, efficient MBP prior to colorectal surgery is broadly used. The purpose of MBP is the preoperative cleansing of the colon from its fecal content. Such a procedure mostly leads to better handling of the bowel intraoperatively. It is also used with the aim of reducing the risk of postoperative complications, such as surgical site infections (SSIs) [63,64,65]. However, several researchers have supported the assertion that MBP did not have a significant impact on the incidence of AL [66,67,68,69]. In 2018, Rollins et al. [70] conducted a study on 21,568 patients who underwent elective colorectal surgery and revealed that MBP did not influence the postoperative complication rate.

Nevertheless, recent evidence suggests that a combination of MBP and preoperative antibiotic administration could contribute to significantly reduced complication rates, including the development of ALs, SSIs and intra-abdominal infections [64,65,71,72]. In a current study, Garfinkle et al. [73] analyzed 40,446 patients undergoing elective colorectal surgery. They found that oral antibiotic preparation alone was associated with lower rates of postoperative complications (i.e., SSIs, ALs, ileus). Interestingly, another research has shown that selective decontamination of the digestive tract (SDD) can reduce the AL rates (7.4% without SDD vs. 3.3% with SDD) [74]. Compared to MBP alone, Grewal et al. [75] found that the addition of oral antibiotic administration to MBP significantly reduced the postoperative AL incidence. The researchers revealed that SDD was associated with significantly lower AL rates in comparison with the preoperative use of broad-spectrum oral antibiotics.

In agreement with these findings, some authors also observed fewer readmissions and decreased AL and SSI rates in patients with oral antibiotic bowel preparation [72]. However, according to the results from the MOBILE trial, mechanical and oral antibiotic bowel preparation did not reduce the AL rates following right or left colectomy (OR, 1.05; 95% CI, 0.15–7.58; *p* = 1.000, or OR, 0.75; 95% CI, 0.19–2.90; *p* = 0.742, respectively) [76].

### 2.2. Intraoperative Risk Factors

Several intraoperative factors appear to influence the risk of developing anastomotic dehiscence after colorectal surgery. Some common ways to evaluate the integrity of intestinal anastomoses and detect ALs during surgery are air leak and dye testing, intraoperative endoscopy and assessment of perfusion at the anastomotic site [49,77]. To date, surgeons are primarily focusing on promptly identifying intraoperative risk factors in order to prevent anastomotic healing complications.

#### 2.2.1. Anastomotic Level

The level of anastomosis and tumor distance from the anal verge are considered significant predictive factors for the development of ALs. Thus far, several researchers have supported the assertion that low colorectal anastomoses are associated with an increased AL risk [15,78,79,80,81]. Indeed, Zhang and colleagues [80] found that tumors located ≤7 cm from the anal verge were correlated with higher postoperative AL rates (OR, 3.445; 95% CI, 1.631–7.279; *p* = 0.001). At the same time, Hamabe et al. [54] also supported the assertion that having a tumor distance from the anal verge less than 7 cm was a significant risk factor for anastomotic dehiscence upon multivariate analysis (*p* = 0.0039). In agreement with results previously reported in the literature [15,79], Kim et al. [81] demonstrated that the tumor location in the middle or lower rectum was an independent risk factor for ALs (*p* = 0.013). A large cross-sectional study by Borstlap et al. [56] found that tumors located ≤3 cm from the anorectal junction were associated with ALs (OR, 1.88; 95% CI, 1.02–3.46).

A systematic review and meta-analysis by Pommergaard [27] showed that distal rectal anastomosis was associated with increased AL risk after CRC surgery. In another study, clinical ALs were presented more frequently in extraperitoneal than intraperitoneal anastomoses (*p* < 0.0001) [82]. Furthermore, Choi et al. [78] revealed that the level of anastomosis ≤5 cm from the anal verge was significantly associated with developing ALs (OR, 6.855; 95% CI, 1.271–36.964; *p* = 0.025). According to Rullier et al. [83], the postoperative AL risk was 6.5 times higher for anastomoses located ≤5 cm from the anal verge than those located >5 cm. Therefore, creating a diverting stoma is highly recommended in cases with low rectal anastomoses in order to reduce the AL risk. 

#### 2.2.2. Protective Stoma

The construction of a diverting stoma (DS) following low anterior resection of rectal cancer patients has been a heated debate and subject of widespread discussion among researchers over the past few decades. Indeed, some authors suggested that creating a protective stoma did not affect the risk of developing postoperative ALs [84,85,86]. However, most studies support that a diverting stoma is highly recommended in cases with distal rectal anastomosis [30,87,88,89,90].

In 2014, Bakker et al. [30] revealed that a temporary protective stoma could be a protective factor for preventing ALs based on multivariate analysis. In another study, constructing a defunctioning stoma was significantly associated with lower AL incidence for distal and proximal rectal cancer regardless of sex [87]. Meanwhile, a prospective randomized multicenter trial showed that the absence of a DS was significantly associated with ALs (*p* = 0.0092) [90]. The authors suggested creating DS in cases, particularly males, with anastomoses below 6 cm.

Furthermore, in a recent meta-analysis involving 8002 patients, Gu et al. [88] concluded that constructing a DS could significantly reduce the postoperative AL and reoperation rates afterection for rectal cancer patients (pooled risk ratio (RR), 0.47; 95% CI, 0.33–0.68; *p* < 0.0001, and RR, 0.36; 95% CI, 028–0.46; *p* < 0.00001, respectively). Chen et al. [89] also supported the assertion that a DS could help lower the incidence of anastomotic dehiscence and re-intervention due to ALs following low anterior resection (RR, 0.34; 95% CI, 0.22–0.53; *p* < 0.00001, and RR, 0.27; 95% CI, 0.16–0.48; *p* < 0.00001, respectively).

Additionally, Gastinger et al. [91] showed that creating a DS could reduce the rate of postoperative ALs after rectal cancer resection that required surgery. Similarly, Hüser et al. [92] revealed that DS was significantly associated with fewer reoperations due to AL complications (*p* < 0.0001). In fact, most authors suggest using a DS following low anterior resection as an effective method to mitigate the clinical consequences of ALs, minimizing the need for surgical re-intervention [88,93,94]. In 2021, Holmgren et al. [95] revealed that creating a defunctioning stoma could affect the absolute permanent stoma risk, with a higher relative risk for patients undergoing partial rather than total mesorectal excision. Regardless of the extent of mesorectal excision, the use of a DS reduced the risk of developing symptomatic AL.

Interestingly, several researchers pointed out that the selective use of a DS should be preferred in patients at high risk for developing ALs [15,54]. Nevertheless, it is not always possible to accurately predict anastomotic dehiscence after low anterior resection [15]. Therefore, the main question remains whether creating a protective stoma is equivalent to overtreating the patients. 

Regarding the most frequently used type of DS (ileostomy over colostomy), temporary loop ileostomy (LI) is mainly preferred. LI is associated with a lower risk of complications, such as stoma prolapse and wound infection, leading to better clinical outcomes [96,97]. However, stoma-associated morbidity and stoma reversal-associated complications should be thoroughly considered, particularly in elderly patients [98].

According to a recent meta-analysis by Gavriilidis et al. [99], stoma prolapse was found at higher rates after a loop transverse colostomy compared to a LI. In fact, patients with colostomies presented with more complications associated with stoma reversal (e.g., wound infections, incisional hernias), whereas patients with ileostomies presented with an increased incidence of high output stoma-related complications. Furthermore, constructing a LI could cause electrolyte and fluid losses, leading to volume depletion and kidney disease [100]. Therefore, increased awareness that kidney disease could be a potential result of LI formation is necessary in order to achieve better outcomes.

#### 2.2.3. Hand-Sewn vs. Stapled Anastomoses

To date, several researchers have focused on comparing the outcomes, including the incidence of AL, between hand-sewn and stapled anastomoses following colorectal surgery. Some authors found no significant difference between AL rates in the hand-sewn over stapled anastomotic technique [87,101]. However, Choy et al. [102] supported in their systematic review that, after ileocolic anastomoses, the AL incidence was significantly lower for stapled end-to-end ileocolic anastomosis than hand-sewn anastomosis. In another study, lower AL rates were also found in patients using the stapled anastomotic technique, particularly end-to-side, compared to the hand-sewn method for ileocolic anastomoses [103].

Contrary to these findings, other researchers revealed that constructing a stapled rather than a hand-sewn anastomosis was associated with increased AL incidence (*p* = 0.03) [104]. Frasson et al. [105] supported the assertion that stapled anastomosis is considered an independent risk factor for developing anastomotic dehiscence after right colectomy based on multivariate analysis (OR, 2.1; 95% CI, 1.1–4.2; *p* = 0.03). Similarly, another study reported a 2-fold increase in AL rates following the stapled versus hand-sewn technique for ileocolic anastomosis [106]. Meanwhile, compared to the handsewn technique, an observational study also revealed that stapled anastomosis after right hemicolectomy was associated with higher AL risk (OR, 1.43; 95% CI, 1.04–1.95; *p* = 0.03) [104]. 

When constructing an intestinal anastomosis, the development of postoperative ALs due to technical issues still remains a feared complication. However, it could be avoided by gaining a better knowledge of certain technical aspects, including the optimal precompression time before firing and compression pressure for secure stapling in colorectal surgery [107,108]. In fact, most authors found that multiple stapler firings affect the AL rates. According to recent research, factors including male gender, rectal carcinoma, neoadjuvant therapy, laparoscopic approach and prolonged operative time (>200 min) were associated with the use of more than one cartridge [109]. Braunschmid et al. [109] revealed that the large diameter of the wall in the middle and lower parts of the rectum as well as the confined space and angle in the laparoscopic approach, are significant risk factors for using two or more cartridges. The prolonged operative duration was also associated with the use of more stapler firings and more complex and challenging surgical procedures. 

In 2011, Kayano et al. [84] showed that multiple firing, particularly using two or more cartridges for rectal transection, was associated with an increased incidence of AL (OR, 3.0589; 95% CI, 1.1–9.5; *p* = 0.0222). Recently, Balciscueta et al. [110] conducted a systematic review and meta-analysis involving 1267 patients with rectal cancer. They supported the assertion that during rectal transection, the need for two stapler firings was considered a risk factor for AL development (OR, 2.44; 95% CI, 1.34–4.42; *p* = 0.003). Other researchers also found that during distal rectal division, three or more stapler firings were associated with a higher risk of AL after laparoscopic rectal resection (OR, 4.6; 95% CI, 1.1–19.2; *p* = 0.03) [111]. Similarly, Park et al. [15] confirmed the association between multiple linear stapler firings and increased AL incidence following laparoscopic rectal resection.

Based on univariate analysis, Kawada et al. [112] found that the number of cartridges (≥3) was associated with higher AL rates when constructing colorectal anastomosis with the double-stapling technique for rectal cancer patients (*p* = 0.041). In particular, upon multivariate analysis, the compression time before stapler firings also affected the risk for developing ALs (OR, 4.58; 95% CI, 1.22–17.20; *p* = 0.024). In agreement with these findings, several researchers supported the significance of the compression time before firing and recommended reducing the number of cartridges used for rectal transection [81,107,112]. Additionally, the application of fibrin glue on stapled colorectal anastomoses, despite its sealing effects, was not found to be correlated with a decreased risk of ALs after laparoscopic rectal cancer resection [79].

#### 2.2.4. Anastomotic Perfusion and Vascular Ligation Level

**Figure 2:**
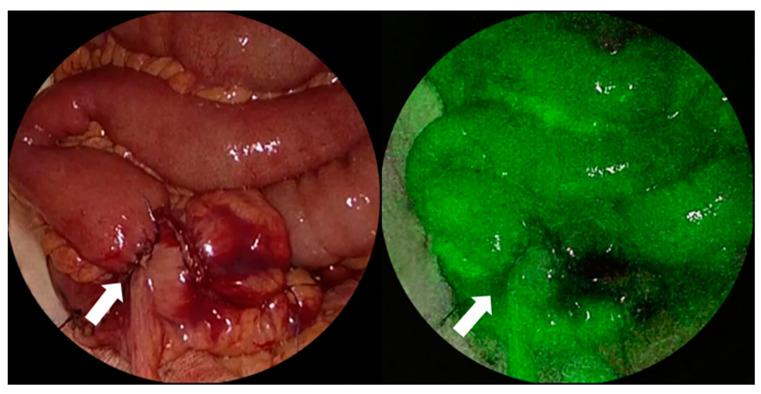
Intraoperative assessment of anastomotic perfusion using ICG-FA in order to construct a well-vascularized anastomosis (white arrows) and reduce the risk of postoperative AL. ICG-FA, indocyanine green-fluorescence angiography; AL, anastomotic leakage.

Interestingly, creating a well-vascularized and technically safe anastomosis without tension is the key to reducing postoperative AL rates and constructing a successful intestinal anastomosis [113]. Indeed, besides the technical issues mentioned above, ensuring sufficient blood supply to the anastomotic site is vital for enhancing intestinal anastomotic healing [113]. Therefore, more researchers have focused on the intraoperative assessment of anastomotic perfusion (Figure 2) and the effects of left colic artery (LCA) preservation on the development of ALs after colorectal surgery [21,114,115,116,117,118,119,120,121,122].

Vignali et al. [114] performed a prospective study on 55 patients undergoing elective curative resection of histologically confirmed rectal or distal sigmoid cancer. They used a laser-Doppler flowmetry technique to evaluate the intestinal blood flow before bowel manipulation and after vascular ligation and bowel transection. They concluded that the reduction in the blood flow at the rectal stump was correlated with a higher risk of developing ALs.

In addition, Alekseev et al. [21] suggested using intraoperative indocyanine green-fluorescence angiography (ICG-FA) to assess anastomotic perfusion. They showed that ICG-FA was an independent factor associated with lower postoperative AL rates (OR, 0.4; 95% CI, 0.2–0.8; *p* = 0.02). In combination with other parameters, ICG-FA may help predict the individual risk of developing ALs. Meanwhile, other authors also confirmed that ICG-FA was associated with a decreased risk of AL and could be an effective tool to predict anastomotic dehiscence after laparoscopic low anterior resection [115,116]. In a retrospective study, Ohya et al. [117] found that based on fluorescence abnormalities, near-infrared imaging using ICG helped change the transection line, reducing the risk of ALs following laparoscopic colectomy. 

Contrary to these findings, a multicenter randomized controlled trial showed that ICG-FA could help in order to evaluate anastomotic perfusion. However, it had no significant impact on AL rates after laparoscopic colorectal resection [123]. Similarly, a multicenter randomized controlled parallel study by Jafari et al. [124] supported the assertion that there was no difference in AL incidence between patients with and without perfusion assessment.

In their study, Hinoi et al. [118] analyzed the results of LCA preservation on AL incidence after laparoscopic anterior resection for middle and low rectal cancer. Their findings revealed a significant association between LCA preservation and low AL rates based on univariate (*p* = 0.005) and multivariate analysis (*p* < 0.001). In fact, regarding the subgroup of patients who underwent en bloc radical lymph node excision (*n* = 411), LCA preservation was also associated with reduced AL rates (*p* = 0.024 and 0.005 upon univariate and multivariate analysis, respectively). LCA preservation after anterior resection may result in adequate blood supply for intestinal anastomosis, even in the 5% of the objects with an absent marginal artery in the left colic flexure [118,119]. In another research, Choi et al. [78] conducted a study on 156 patients who underwent laparoscopic rectal surgery without constructing a diverting ileostomy. However, they found no significant association between inferior mesenteric artery (IMA) ligation level and the risk of developing ALs.

With the aim of evaluating the relationship between high arterial ligation and AL rates after anterior resection, a population-based study revealed that the use of a high tie was not correlated with the risk of developing postoperative AL (OR, 1.00; 95% CI, 0.72–1.39) [125]. Meanwhile, a meta-analysis of randomized controlled trials by Kong et al. [126] showed that high ligation of the IMA did not influence the AL incidence following anterior resection for rectal cancer patients. In fact, the relationship between the development of AL and the level of IMA ligation still remains a subject of debate after distal sigmoid and rectal cancer resection [120,121,122].

A prospective cohort study was conducted using laser-Doppler flowmetry in order to assess the impact of the level of arterial ligation on the colonic limb perfusion and the extent of the mesorectal excision on rectal blood flow [127]. The authors found that the high tie ligation did not reduce the perfusion of the colonic limb. At the same time, the mean rectal blood flow ratio was lower for patients undergoing total rather than partial mesorectal excision. Depending on tumour height, a total or partial mesorectal excision might be performed [128]. Back et al. [128] revealed an elevated AL risk for cases with low anastomoses, probably due to the blood perfusion compromise. Compared to partial mesorectal excision, the patients who underwent total mesorectal excision were found with reduced blood flow in the posterior rectal quadrant, eventually leading to higher AL risk.

#### 2.2.5. Laparoscopic vs. Open and Robotic Colorectal Surgery

Laparoscopic surgery is gaining ground as a safe and feasible technique for treating CRC patients when performed by skilled surgeons. Besides some technical challenges, mainly concerning male, obese patients with a narrow pelvis, the advantages of laparoscopic colorectal surgery have been broadly acknowledged. Furthermore, a typical example of the characteristic differences between laparoscopic and open procedures is the need for multiple stapler firings during laparoscopic low anterior resection performed for rectal transection, and also commonly correlated with increased AL risk [10,129]. Nevertheless, this risk of developing ALs could be decreased through recent advances in stapling technology [10,129].

A randomized trial revealed no association between open or laparoscopic surgery and the risk of developing postoperative ALs for rectal cancer patients [130]. In agreement with these results, other studies also found no significant difference between open and laparoscopic procedures for rectal resection regarding the AL rates [131,132,133]. In addition, some authors support the assertion that conversion from laparoscopic to open colorectal surgery did not affect AL incidence [134], whereas others noted a significant association between conversion and AL rates [135,136] and revealed a 7.9-fold greater risk for ALs in patients who underwent conversion of laparoscopic anterior resection to open surgery [136]. 

Transanal total mesorectal excision (TaTME) represents a promising surgical approach for rectal cancer patients. However, further studies are required to evaluate the postoperative risk of complications, particularly ALs [49]. CRC treatment enters a new era with significant improvements and technological advances resulting in the evolution of robotic surgery. Even though the benefits of robotic colorectal surgery are accepted [137], studies have shown no advantages over laparoscopic surgery regarding post-surgical complications [138,139]. In fact, some researchers found comparable AL rates between the laparoscopic and robotic approaches [140,141].

#### 2.2.6. Pelvic and Transanal Drain Placement

The relationship between AL development and prophylactic anastomotic drainage after CRC surgery constitutes a subject of ongoing debate. Unfortunately, there is still insufficient evidence regarding the role of pelvic drainage in AL prevention [142]. Indeed, Denost et al. [143] conducted a multicenter randomized trial to evaluate the benefits of prophylactic pelvic drainage following rectal cancer surgery. They found that the rates of postoperative pelvic sepsis were similar between patient groups with and without pelvic drains (*p* = 0.58). Based on univariate analysis, another research team revealed that AL rates were lower in the patient group with pelvic drainage than in the group without drains, but without reaching statistical significance after laparoscopic low anterior resection (*p* = 0.18) [112]. 

However, upon multivariate analysis, Akiyoshi et al. [86] found that the lack of postoperative pelvic drain placement was considered an independent predictive factor for AL development in rectal cancer patients (OR, 3.814; 95% CI, 1.207–12.644; *p* = 0.0225). Additionally, another study also showed that the placement of a pelvic drain was associated with decreased risk of anastomotic dehiscence following laparoscopic anterior resection (OR, 0.43; 95% CI, 0.19–0.94; *p* = 0.04) [144]. Other researchers found that using at least one drain was significantly associated with a mild clinical course among rectal cancer patients with ALs, mostly without necessitating surgical management [87]. 

A systematic review and meta-analysis showed that pelvic drainage was associated with lower rates of anastomotic failure (OR, 0.51; 95% CI, 0.36–0.73) and reintervention (OR, 0.29; 95% CI, 0.18–0.46) in rectal cancer patients following anterior resection and the construction of an extraperitoneal colorectal anastomosis [145]. Nevertheless, a meta-analysis of randomized controlled trials involving 660 patients who underwent rectal surgery with extraperitoneal anastomosis revealed no significant impact of pelvic drain placement on AL rates after rectal resection [146]. Similarly, another meta-analysis of four randomized controlled trials involving 760 patients also supported the assertion that the routine use of pelvic drains did not affect the postoperative AL incidence following rectal surgery (OR, 0.99; 95% CI, 0.65–1.49; *p* = 0.95) [147].

Meanwhile, the absence of a transanal decompression tube was considered an independent risk factor for developing ALs among patients with stage 0/I rectal cancer after laparoscopic low anterior resection (OR, 3.11; 95% CI, 1.01–9.524; *p* = 0.0484) [148]. In fact, Ito et al. [85] supported in their retrospective study that the placement of a transanal tube could prevent AL development. The absence of the transanal tube appeared to be an independent risk factor for anastomotic dehiscence following laparoscopic low anterior resection (OR, 33.5; 95% CI, 1.82–618; *p* = 0.018). In contrast, Hamabe et al. [54] found that anal drainage did not influence the incidence of AL (*p* = 0.3399).

#### 2.2.7. Timing of CRC Surgery (Elective vs. Emergency)

Colorectal ALs are primarily detected in patients undergoing emergency surgery for peritonitis or bowel obstruction rather than elective colorectal surgery [10,149]. Hemodynamic instability and/or hypoalbuminemia without preoperative mechanical and oral antibiotic bowel preparation represent only a few significant factors that may have a negative impact on any anastomosis, eventually leading to anastomotic failure [10,149].

#### 2.2.8. Operative Time

Recent studies have shown the impact of prolonged operative time on the risk of developing postoperative ALs [48,78,79,150]. In their research, Choi and colleagues [78] found that the operative time was significantly associated with ALs (OR, 8.115; 95% CI, 1.982–33.222; *p* = 0.004) following laparoscopic resection for rectal cancer. Meanwhile, another research revealed that prolonged operative duration (>220 min) was independently associated with the development of ALs after laparoscopic rectal cancer surgery upon multivariate analysis [79]. Overall, longer operative time than 3 h has been associated in several studies with an increased risk of developing anastomotic dehiscence [48,150].

To our knowledge, prolonged operative time may indicate more complex or challenging surgical procedures, which are already associated with a high expected risk of postoperative complications. Apart from the operative difficulty, the increased surgical duration could affect the complication risk through various factors, including surgeon fatigue. On the other hand, surgical training may sometimes lead to longer operative times. Surgical speed is commonly related to several factors, including a higher risk for intraoperative errors. Therefore, the surgical team should not sacrifice their performance and efficiency in order to shorten the operative time. 

#### 2.2.9. Surgeon’s Experience

In general, a surgeon’s experience matters when it comes to achieving better patients’ outcomes and minimizing the risk of complications. In an effort to evaluate the impact of a surgeon’s experience on AL rates, some researchers have shown that the individual surgeon is a risk factor for developing ALs (*p* < 0.0001) [151,152]. Therefore, colorectal specialization is considered an effective way to prevent postoperative complications, including anastomotic failure [153].

In another study, Kayano et al. [84] found that experienced surgeons had lower AL rates after laparoscopic low anterior resection. Nevertheless, there was no significant difference between the surgeon case volume and the risk of postoperative ALs. Meanwhile, Park et al. [15] supported the assertion that there was no significant association between AL incidence and each surgeon’s prior experience of total mesorectal excision. Finally, a systematic review and meta-analysis conducted by Kelly et al. [154] showed that trainees had lower AL incidence (*p* = 0.01). However, no difference was observed between expert surgeons and supervised trainees (*p* = 0.08).

### 2.3. Perioperative Risk Factors

Most perioperative risk factors usually linked with anastomotic failure appear to be associated with the administration of medications, compositional alterations in gut microbiota and blood loss. In fact, several ongoing studies aim to investigate the impact of these factors on postoperative AL development.

#### 2.3.1. Pharmaceutical Therapy

Long-term corticosteroid use was found to be related to increased AL rates [28,150,155]. Another research showed that prolonged and perioperative use of corticosteroids was also correlated with elevated AL risk [36]. Contrary to some studies [156,157], other researchers observed a significant association between the use of non-steroidal anti-inflammatory drugs (NSAIDs) and a higher risk of developing postoperative ALs [158].

Furthermore, some authors pointed out an association between increased AL risk and immunosuppressive drugs, such as mycophenolate mofetil, commonly administrated for patients after organ transplantation [38,159]. Regarding other immunosuppressive agents, including cyclosporine A, tacrolimus and everolimus, experimental evidence reveals higher AL rates [160,161,162]. In addition, bevacizumab, a vascular endothelial growth factor inhibitor, should be considered before its administration since it may impair the anastomotic healing following colorectal surgery [163]. Additionally, preoperative infliximab therapy was not significantly associated with AL incidence after laparoscopic colorectal surgery for irritable bowel disease (*p* = 0.81) [164].

#### 2.3.2. Gut Microbiome

During the last decade, accumulating evidence suggests that gut microbiota and its perioperative compositional alterations may play a key role in the process of anastomotic healing following colorectal surgery [165]. Shogan et al. [166] conducted an experimental study and revealed a significant association between Enterococcus faecalis, a common commensal bacterium colonizing the gastrointestinal tract of healthy humans, and AL pathogenesis. In fact, they showed that Enterococcus faecalis could lead to AL development due to its ability to degrade collagen and activate host intestinal matrix metalloproteinase 9 (MMP9). The researchers suggested that intestinal microbes with high collagen-degrading/MMP9-activating activity might be associated with AL pathogenesis. Despite the multifactorial pathophysiology of ALs, gut microbiota might influence the anastomotic integrity postoperatively [167]. However, further investigation is still required to elucidate the potential underlying mechanisms that could lead to impaired anastomotic healing and, eventually, anastomotic dehiscence.

In 2015, a research team found that increased AL incidence was associated with Lachnospiraceae abundance and low microbial diversity levels [168]. In fact, the abundance of the Lachnospiraceae family was also positively correlated with BMI. A few years later, the researchers isolated bacterial DNA from 123 “donuts” of patients who underwent stapled colorectal anastomosis [169]. The C-seal was used in the stapled anastomoses of 63 patients. In non-C-seal samples, low microbial diversity and a high abundance of mucin-degrading members of the Bacteroidaceae and Lachnospiraceae families were linked to increased AL incidence. In contrast, the development of ALs was not associated with intestinal microbiota composition in C-seal patients. Overall, additional clinical evidence is necessary to evaluate the role of the gut microbiome in the loss of tissue integrity at the anastomotic site [167]. 

#### 2.3.3. Other Perioperative Complications and Events

Several researchers have revealed the importance of perioperative blood loss and transfusion in raising the incidence of ALs. For example, in 2014, some authors found that based on univariate analysis, the intraoperative bleeding volume of ≥100 mL was associated with increased AL rates (*p* = 0.037) [112]. In addition, preoperative hemoglobin levels <11 g/dL were associated with anastomotic dehiscence [170,171], having a 6.5-fold increased risk of developing ALs [171]. 

Other researchers have found a positive correlation between perioperative bleeding and AL risk [29,152,172,173,174]. Notably, Marinello et al. [152] supported the assertion that the need for perioperative blood transfusion was independently associated with AL development following colon cancer resection (OR, 2.83; 95% CI, 1.59–5.06; *p* < 0.0001). Similarly, Lai et al. [174] revealed a significant association between increased perioperative blood transfusion and AL rates after anterior resection (*p* < 0.001). They also supported the assertion that the incidence of ALs could be affected by anesthesia duration on multivariate analysis (*p* = 0.033) and intraoperative hypotension on univariate analysis (*p* = 0.022).

## 3. Clinical Presentation

The clinical presentation of ALs may include a wide range of symptoms, from subtle and non-specific to more severe ones. Indeed, cardiovascular symptoms (e.g., cardiac arrhythmias), fever, pain and purulent drainage or signs of peritonitis might be the clinical manifestations of ALs [175]. According to Boström et al. [176], moderate or severe pain after surgery was related to a higher postoperative AL risk (OR, 1.69; 95% CI, 1.21–2.38). In fact, the researchers suggested that severe pain on the first postoperative day should also raise suspicion for AL development. Therefore, increased awareness of this dreadful complication is crucial for surgeons in order to detect early signs of postoperative ALs.

Overall, ALs commonly occur within the first two weeks after colorectal surgery [8,9]. In fact, most ALs are diagnosed 7 to 12 days postoperatively [8,177]. Hyman et al. [8] showed that several patients (42%) were diagnosed after their discharge from the hospital, and others (12%) were diagnosed after the 30th postoperative day. A cross-sectional study revealed that one-third of ALs was diagnosed after the 30th postoperative day following low anterior resection [56]. In fact, approximately half of the ALs did not heal, leading to an overall proportion of chronic presacral sinus of 9.5% [56]. Early ALs usually develop during the first 30 postoperative days [178,179], while very early ALs usually develop within the first five days after surgery [180]. In contrast, other researchers suggested that early and late ALs occur until and after the first six postoperative days, respectively [180]. Consequently, since there has yet to be a consensus among surgeons about the early and late AL definition, it is necessary to find the appropriate cutoff point for accurate discrimination between them. Interestingly, an early AL mostly occurs as an anastomotic failure due to surgical and surgeon-related factors [181]. However, late ALs are commonly associated with patient-related factors, particularly deficiencies concerning the process of anastomotic healing [181].

A retrospective observational cohort study by Jutesten et al. [182] revealed that patients with postoperative AL were more prone to present with major low anterior resection syndrome than patients without any postoperative AL. Furthermore, the authors found that ALs were significantly associated with the major low anterior resection syndrome and the symptom of urgency postoperatively (relative risk 2.3; 95% CI, 1.4–3.9, and relative risk 2.1; 95% CI, 1.1–4.1, respectively). Based on multivariate analysis, ALs were correlated with increased mortality rates in cases when reintervention was performed (OR, 5.57; 95% CI, 3.29–9.44) [24]. A notable mortality increase was found in the elderly presenting with postoperative anastomotic failure [24]. Regarding the permanent stoma prevalence after anterior resection, Holmgren et al. [183] supported the assertion that stoma permanence is common, whereas stoma reversal surgery increases the risk of severe complications.

## 4. Diagnostic Workup

A high level of suspicion is vital for the early diagnosis of ALs, leading to prompt medical attention and increased treatment effectiveness. Indeed, delayed diagnosis is mainly linked to poor patient outcomes, affecting AL treatment initiation. Patients presenting with postoperative ALs are closely monitored for clinical signs of peritonitis and increased inflammatory markers. In general, using these markers as a part of the diagnostic process is considered an effective tool for the early detection of ALs. Elevated serum levels of an acute-phase reactant, C-reactive protein (CRP), are commonly found in patients experiencing complications after CRC surgery [12]. In fact, CRP and white blood cell (WBC) count are used for evaluating postoperative intra-abdominal inflammation [12]. In addition, CRP and procalcitonin levels should be closely monitored following colorectal surgery and be considered in case further diagnostic imaging tests would be necessary [184]. 

Computed tomography (CT) scan of the abdomen remains a broadly used imaging technique for AL diagnosis. The timing of postoperative imaging, the CT image quality and the experience of radiologists are significant factors that affect the diagnostic accuracy of abdominal CT scans [184]. However, the diagnostic performance of this examination for AL diagnosis can be significantly improved by using a water-soluble contrast enema [160]. Such methods could lead to the detection of postoperative ALs, reaching 100% sensitivity in identifying this severe complication [185]. Compared with oral contrast, the possibility of rectal contrast enema reaching the anastomotic site is significantly higher [186]. At the same time, the predictive value of a rectal-contrast CT scan is also increased for AL diagnosis after colon and rectal surgery [186]. Therefore, according to several researchers, the addition of rectal contrast in abdominal CT scans should be considered the diagnostic procedure of choice for ALs after CRC surgery [185,187].

### Scoring Systems and Other Predictive Biomarkers of Anastomotic Leakage

So far, several scoring and grading systems for ALs have been described in the literature. With the aim of predicting and severity grading of ALs, these systems are useful for the risk assessment of anastomotic failure and further guidance of surgeons on the optimal treatment options. The colon leakage score (CLS), composed of different clinical parameters, was suggested by Dekker et al. [188] as an effective tool to predict ALs in patients undergoing left-sided colorectal surgery. Taking into consideration the score, CLS could guide the surgeons in constructing either an intestinal anastomosis or a nonfunctional stoma. Based on five variables, the modified CLS, which was initially developed by Yang et al. [189], could perform better than the CLS for AL prediction (*p* = 0.008).

In addition, the Dutch leakage (DULK) score (composed of 13 parameters) and the modified DULK score (including patient’s clinical condition, abdominal pain not related to the wound, respiratory rate and CRP levels) are also used for assessing the risk of anastomotic failure [190]. However, the modified DULK score is more useful in clinical practice since it comprises fewer parameters than the original score. Regarding the modified DULK score, with at least one present parameter, the overall sensitivity, specificity and negative predictive value (NPV) were 97%, 57% and 99.5%, respectively [190].

Following meticulous research on the literature, the ISREC defined AL and proposed three severity grades of this complication, considering their impact on the patients’ management after anterior resection of the rectum [6]. Grade A leakage does not require any intervention. However, grade B leaks require therapeutic (not surgical) intervention, whereas grade C ALs mandate relaparotomy. Regarding colorectal ALs, an international expert panel consisting of colorectal surgeons suggested that the grading system mentioned above should be complemented with the Clavien–Dindo classification of postoperative complications [7].

In another study, Hu et al. [191] recommended a prediction model for ALs after laparoscopic total mesorectal excision based on four parameters, including sex, history of diabetes, level of anastomosis and intraoperative bleeding. Similarly, another scoring system was also developed by Han et al. [192] in order to predict the risk of ALs, taking into account six parameters (i.e., gender, history of preoperative chemoradiation, tumor diameter, operation time, intraoperative blood loss and level of anastomosis).

Furthermore, Shiwakoti et al. [193] proposed a prediction model for ALs in patients undergoing laparoscopic surgery for rectal cancer based on different variables (i.e., BMI, tumor size, operation time, history of diabetes mellitus and preoperative chemoradiotherapy). Recently, Hoek et al. [194] suggested using a preoperative prediction model for anastomotic failure, evaluating various parameters, including gender, age, BMI, ASA physical status classification, history of neo-adjuvant (chemo)radiotherapy, cT stage, tumor distance from the anal verge, and deviating ileostomy. However, further well-designed studies are still required in order to validate these innovative methods for predicting ALs. Indeed, the integration of such prediction models in clinical practice could aid surgeons in optimal intraoperative decision-making and patient counselling.

In 2020, a research team supported the assertion that lower CRP levels after the second postoperative day and serum CRP levels <180 mg/L on the fourth postoperative day could be used as a reliable method to exclude AL diagnosis [195]. However, another study involving 129 patients after laparoscopic colorectal surgery revealed that CRP and WBC count were poor predictors of ALs and other septic complications [196]. 

In a systematic review and meta-analysis involving 2483 patients, Singh et al. [197] showed that the measurement of CRP levels on postoperative days 3 to 5 was an accurate negative predictive test, determining those patients that were unlikely to present with ALs following colorectal surgery. Another research supported the assertion that CRP and, mainly, procalcitonin levels were accurate at predicting major ALs (necessitating drainage or reintervention), with a high negative predictive value on postoperative days 3 to 5 [198]. Consequently, evaluating CRP and procalcitonin concentrations on these postoperative days could assist the surgeons in clinical decision-making before the patients’ discharge.

In their study, Jin et al. [199] showed that CRP was an accurate predictor of ALs after laparoscopic rectal surgery. Raised serum CRP levels on postoperative days 4 to 7 were associated with the need for more careful evaluation of patients. Other researchers suggested that the postoperative CRP concentrations below 44 mg/L and 27.2 mg/L on the second and fourth postoperative days were highly sensitive in excluding AL [200]. Meanwhile, Reynolds et al. [201] also supported the utility of the measurement of CRP levels as a negative predictive test for anastomotic failure.

A prospective multicentre observational study showed that the DULK score and CRP concentrations were good positive and excellent negative predictors of developing anastomotic dehiscence [202]. The addition of procalcitonin to the assessment of the DULK score and serum CRP levels assisted in predicting or excluding AL diagnosis. Another study supported the assertion that the predictive value of procalcitonin for ALs was improved when combined with CRP and WBC count [203]. At the same time, other researchers revealed high NPVs of CRP and procalcitonin for anastomotic failure in the early postoperative period [204].

Furthermore, a prospective study showed that oxidative stress indicators, particularly malondialdehyde (MDA), in patients’ serum and drain fluid could be a valuable biomarker for early AL diagnosis following rectal cancer surgery [205]. A systematic review also showed that combining peritoneal drain fluid (such as interleukin-6, interleukin-10 and tumor necrosis factor) and systemic (including CRP, WBC count and procalcitonin) biomarkers improved their predictive accuracy for anastomotic dehiscence [206]. Additionally, other researchers found altered protein expression patterns of the inflammatory markers monocyte chemoattractant protein 2 (CCL8/MCP2), leukemia-inhibiting factor (LIF), and epithelial-derived neutrophil-activating protein (CXCL5/ENA-78) in the peritoneal fluid of patients that developed ALs [207]. In 2018, Shimura et al. [208] suggested that low serum albumin levels could be used as an indicator of ALs after surgery for CRC patients. Overall, gaining in-depth knowledge and a better understanding of the risk factors associated with postoperative ALs could help identify those patients who would benefit from constructing a defunctioning stoma [49].

A wide range of systemic or peritoneal drain fluid inflammatory biomarkers has been explored so far for their utility in predicting ALs after colorectal surgery. Indeed, serum and fecal calprotectin levels have been investigated for early AL diagnosis [209,210]. In fact, some authors suggested using the combined assessment of serum or fecal calprotectin and CRP levels in order to achieve a prompt diagnosis of AL development [210,211]. Other researchers analyzed serum, urinary and peritoneal fluid levels of neopterin [212], a product of macrophages commonly associated with autoimmune and infectious diseases, cancer and sepsis [213]. They found significantly elevated preoperative and postoperative urinary neopterin:creatinine ratio in patients that developed ALs compared to those without postoperative complications (*p* = 0.037 and 0.012, respectively) [212].

Previous studies on serum biomarkers, usually associated with various ischemic conditions, have shown a lack of specificity for detecting ALs [214]. Up to date, novel research supported the use of ischemic biomarkers in the peritoneal fluid for the early recognition of ALs. Indeed, the increased peritoneal lactate:pyruvate ratio has been correlated with anastomotic dehiscence in several studies [215,216]. In addition, measuring pH through a catheter positioned near the constructed anastomosis could help predict anastomotic failure. The assessment of anastomotic intramucosal pH within the first 24 h after surgery showed that the risk of developing AL was 22 times higher in patients with a pH < 7.28 (sensitivity 28%, specificity 98%) [217]. Meanwhile, measurements of the pH in peritoneal drain fluid also revealed a significant decrease in patients needing reintervention for their postoperative AL management [218]. Recently, more researchers have been focusing on using sensors placed at the anastomotic site to monitor intestinal tissue oxygen tension (ptO_2_) and pH values, eventually detecting tissue hypoxia and impaired anastomotic healing [219].

According to Reisinger et al. [209], the preoperative measurement of intestinal fatty acid binding protein levels was predictive of AL after colorectal surgery, reaching 50% sensitivity and 100% specificity. Furthermore, another novel research revealed that patients with a polymorphism related to low cyclooxygenase-2 (COX-2) levels are prone to developing AL [220]. In fact, the authors also showed that the lack of COX-2 in mice was associated with increased AL rates, impaired angiogenesis and, eventually, anastomotic healing. Interestingly, rectal cancer patients presenting with AL might have an upregulated inflammatory response preoperatively with increased serum levels of C-X-C motif chemokine 6 (CXCL6) and C-C motif chemokine 11 (CCL11) [221].

Based on CT body composition, Xiang et al. [222] developed and validated a nomogram that, combined with gender, blood glucose, nutrition risk screening, skeletal muscle area and visceral fat area, was a reliable and accurate method for AL prediction. Interestingly, recent studies have shown the critical role of artificial intelligence (AI) systems in improving CRC screening, diagnosis and treatment [223]. Applications of these novel AI-guided techniques showed promising results in predicting CRC surgery complications, especially ALs. Using auto-AI, Mazaki et al. [224] developed and presented an accurate predictive model for ALs after colorectal surgery with double-stapling technique anastomosis. The area under the curve was 0.766. In another research, machine learning-based random forest predicted ALs (area under the curve = 0.87) and provided practical advice on whether to construct a temporary stoma [225]. Additionally, Sammour et al. [226] validated an online risk calculator for postoperative AL prediction and examined AI-based analytics. At the same time, other researchers developed an AI-based real-time microcirculation analysis technique in ICG angiography in order to predict complications of intestinal anastomoses for patients undergoing laparoscopic colorectal surgery [227].

## 5. Conclusions

Overall, AL represents one of the most severe complications following CRC surgery, commonly associated with high morbidity and mortality rates. To date, several researchers have been focusing on identifying all these risk factors that affect the incidence of postoperative ALs. Indeed, gaining a better knowledge and understanding of specific preoperative, intraoperative and perioperative factors could help the surgeons’ intraoperative judgement and decision-making. Thus far, several prediction models and biomarkers for ALs have shown promising results. However, additional studies are still required to shed more light on developing accurate models or biomarkers for predicting this severe complication.

## Data Availability

The data presented in this research are available on request from the corresponding author.
